# Light Induced Changes in Protein Expression and Uniform Regulation of Transcription in the Thylakoid Lumen of *Arabidopsis thaliana*

**DOI:** 10.1371/journal.pone.0005649

**Published:** 2009-05-21

**Authors:** Irene Granlund, Michael Hall, Thomas Kieselbach, Wolfgang P. Schröder

**Affiliations:** 1 Department of Chemistry, Umeå University, Umeå, Sweden; 2 Umeå Plant Science Center, Umeå, Sweden; Gothenburg University, Sweden

## Abstract

In plants oxygenic photosynthesis is performed by large protein complexes found in the thylakoid membranes of chloroplasts. The soluble thylakoid lumen space is a narrow and compressed region within the thylakoid membrane which contains 80–200 proteins. Because the thylakoid lumen proteins are in close proximity to the protein complexes of photosynthesis, it is reasonable to assume that the lumen proteins are highly influenced by the presence of light.

To identify light regulated proteins in the thylakoid lumen of *Arabidopsis thaliana* we developed a faster thylakoid preparation and combined this with difference gel electrophoresis (DIGE) of dark-adapted and light-adapted lumen proteomes. The DIGE experiments revealed that 19 lumen proteins exhibit increased relative protein levels after eight hour light exposure. Among the proteins showing increased abundance were the PsbP and PsbQ subunits of Photosystem II, major plastocyanin and several other proteins of known or unknown function. In addition, co-expression analysis of publicly available transcriptomic data showed that the co-regulation of lumen protein expression is not limited to light but rather that lumen protein genes exhibit a high uniformity of expression.

The large proportion of thylakoid lumen proteins displaying increased abundance in light-adapted plants, taken together with the observed uniform regulation of transcription, implies that the majority of thylakoid lumen proteins have functions that are related to photosynthetic activity. This is the first time that an analysis of the differences in protein level during a normal day/night cycle has been performed and it shows that even a normal cycle of light significantly influences the thylakoid lumen proteome. In this study we also show for the first time, using co-expression analysis, that the prevalent lumenal chloroplast proteins are very similarly regulated at the level of transcription.

## Introduction

Higher plants, algae and cyanobacteria have the ability to perform oxygenic photosynthesis, and in higher plants the process is centred in the thylakoid membrane of the chloroplast. Enclosed by the thylakoid membrane is a space, the thylakoid lumen, which has a limited distance between the membranes of 40–100 Å [1]. The lumen compartment was for a long time believed to function mainly in balancing the ion currents over the thylakoid membrane, however today 80–200 different proteins are suggested to be located there [2], [3]. The small proteome is comprised of a wide range of different types of proteins, some extensively studied while others have unknown function. The most abundant proteins of the lumen are the extrinsic photosystem II (PSII) proteins PsbO, PsbP and PsbQ, and also plastocyanin [2]. The other known proteome members consist of a large group of at least eight immunophilins, a family of PsbP-like proteins, chaperone and proteolytic activity associated proteins, peroxiredoxin Q, two pentapeptide repeat proteins and a number of unclassified proteins such as TL29. Considering the obvious, being the location of the lumen proteins within the thylakoid membrane, the heart of photosynthesis, it would be expected that several if not many of the proteins participate in the regulation of photosynthesis. Indeed among those lumen proteins which have not previously been identified as extrinsic PSII components several have been implicated in PSII function. PPL1, a member of the PsbP-like protein family, as well as the 18.3 kDa lumen protein, have both recently been shown to play roles in the efficient repair of photodamaged PSII [4], [5]. Furthermore, mutant plants containing a T-DNA insertion in the *CYP38* gene are defect in assembly of PSII supercomplexes [6].

Plants have evolved a number of mechanisms for response to changeable environmental conditions, especially to variations in light. On the molecular level light is sensed by photoreceptors such as phototrophins, cryptochromes and phytochromes as well as by redox signals originating from the photosynthetic electron transport chain [7]. In addition to and in concert with these light-sensing mechanisms, plants also use programmed responses commonly named the circadian clock (rhythm). Microarray analysis has shown that the mRNA-expression of genes encoding several subunits of both photosystems are under circadian control [8] and that ∼23% of photosynthesis genes exhibit >1.75 fold diurnal expression changes [9]. In general plants are found to exhibit increased photosynthesis, growth, survival and competitive advantages when there is a synchronisation between the circadian clock and light-dark cycles [10]. Considering the critical role the light-dark cycle plays in the regulation of photosynthesis itself, it is also of importance to increase the scope of our understanding to also include proteins in direct or close proximity with the photosynthetic machinery. To study this we have identified thylakoid lumen proteins displaying light induced abundance changes using difference-gel-electrophoresis (DIGE). DIGE is a proteomics method where fluorescent probes are covalently added to proteins prior to separation by two-dimensional gel electrophoresis [11]. Typically two different samples are labelled by Cy3 and Cy5 probes respectively while an internal standard, containing an equal mix of both samples, is labelled with Cy2. Because both samples are run in the same gel, gel to gel and other experimental variation is minimized. Furthermore the inclusion of an internal standard in each gel makes normalization between different gels possible and facilitates the identification of proteins changing in abundance by statistical methods [12]. With respect to the thylakoid lumen, the original extraction method [13] is time consuming, taking roughly 4–6 hours to perform. This means that all changes that are faster than this will be missed or masked in the analysis. To solve this problem we have here developed a faster lumen isolation method. The original method was focused on obtaining highly pure lumen and identifying truly lumen located protein, whereas we have here shortened this procedure in order to make it faster but at the expense of purity.

As a complement to our study of expression of lumenal chloroplast proteins during the change from darkness to light, we have examined transcriptional profiles of known lumen protein genes using co-expression analysis. With the emergence of large publicly available gene expression databases, co-expression analysis has become a powerful tool for the identification of genes involved in the same or related biological processes [4], [14], [15], [16]. In co-expression analysis one searches for genes that show a similar expression profile across numerous different microarray experiments, representing different environmental and stress conditions as well as developmental stages and tissue types. This analysis method was applied to genes encoding the proteins of the Arabidopsis lumen, both in order to examine if the proteins identified using DIGE show common regulation beyond the response to light, and also to characterise differences and similarities in transcriptional expression among all the known prevalent lumen proteins.

In this work we show, using difference gel electrophoresis (DIGE) on fast lumen preparations, that 21 proteins changed in abundance between 8 h-light and 16 h-dark acclimated Arabidopsis plants. The up-regulated lumen proteins were dominated by proteins connected with photosynthetic performance. Furthermore co-expression analysis of prevalent lumen protein genes reveals that the majority of protein members of the chloroplast lumen are uniformly co-expressed on the transcriptional level.

## Results

### Evaluation of a new fast lumen preparation

**Figure 1:**
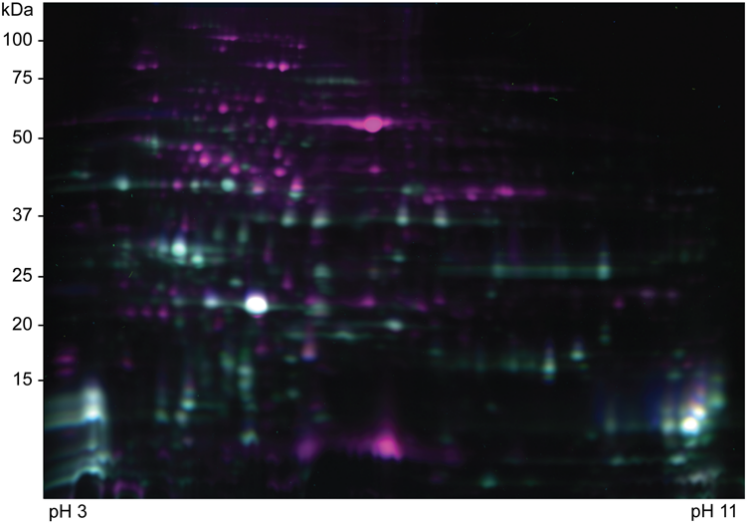
DIGE-gel image showing differences in normal and fast thylakoid lumen preparations. Composite DIGE-gel image of normal and fast thylakoid lumen preparations. Light blue spots represent proteins observed in the normal preparation and purple spots represent chloroplast stroma contaminations specifically observed in the fast preparation.

In order to trap the proteome at a specific metabolic state or situation it is important that the extraction is fast. Therefore the original lumen preparation method for Arabidopsis, developed by [13], which takes 4–6 hours, was modified by omitting thylakoid membrane washing steps (for details see Materials and methods). By doing this the extraction time was reduced to around two hours, however, this unambiguously leads to more stroma protein contamination, as illustrated in figure 1. In this DIGE experiment the purple spots indicate the additional spots observed when performing the fast thylakoid lumen preparation as compared to the normal preparation. The purple-marked proteins in figure 1 were identified by MALDI-TOF MS and it was found that the major part of contamination as expected originated from stromal proteins (data not shown). The experimental design for the experiment is showed in table 1.

**Table 1:** Experimental design for fast vs. normal Arabidopsis thylakoid lumen preparation DIGE experiment.

Gel nr.	Cy 2	Cy 3	Cy 5
1	pooled standard	Normal preparation	16 h dark-adapted A, with NaBr
2	pooled standard	16 h dark-adapted A	Normal preparation
3	pooled standard	8 h light-adapted A, with NaBr	Normal preparation
4	pooled standard	Normal preparation	8 h light-adapted A

### Differences in the lumen proteomes of light- and dark-adapted Arabidopsis plants

The lumen space is very narrow, and some of the thylakoid membrane located protein complexes (PSII, Cytochrome b_6_/*f*) extend into the lumen. It is logical to assume that most proteins in this compartment are in close proximity to each other and to the light-regulated photosynthesis complexes in the membrane. Thus it is reasonable to assume that also the lumen located proteins may be influenced by light. This is further corroborated by examination of publicly available microarray data using the Arabidopsis eFP Browser (bar.utoronto.ca, Short Day series, [17], [18]). Analysis of data from Arabidopsis seedlings, grown in 8 h light/16 h dark cycles under comparable growth conditions as used in this study shows that mRNA expression for the majority of prevalent lumen proteins cycles in a diurnal manner, with expressions peaking during the light period (data not shown).

**Figure 2:**
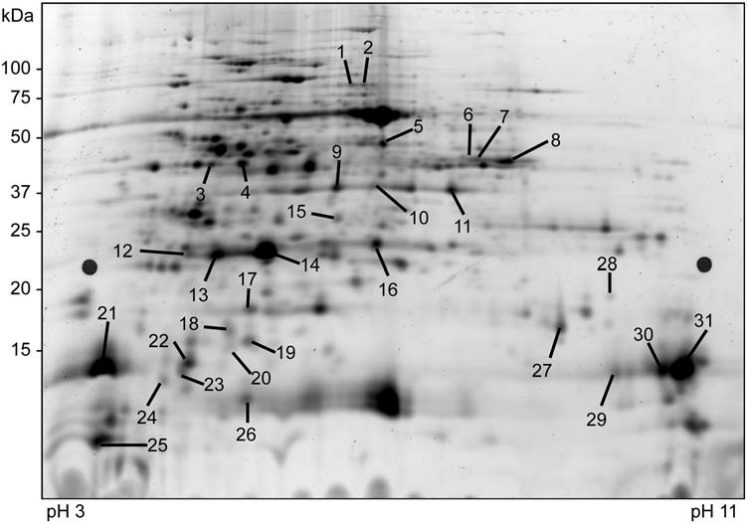
Representative 2D-gel image of 8 h light-adapted and 16 h dark-adapted thylakoid lumen proteomes of *Arabidopsis thaliana.* Protein spots displaying significant changes in spot intensity between treatments (marked by assigned numbers) were excised, in-gel digested with trypsin and identified by peptide mass fingerprinting.

To determine if this also holds true at the protein level and in that case to which extent, light induced expression changes were studied using difference-gel-electrophoresis (DIGE), comparing the contents of the thylakoid lumen of *Arabidopsis thaliana* after a dark period (16 h dark-adapted plants) and a light period (8 h light-adapted plants). Chloroplast lumen was prepared from seven week old light-adapted and dark-adapted Arabidopsis plants using the fast lumen protocol. Four independent fast lumen preparations were used in a dye-swap experimental design as described in table 2. In all gels a Cy2 labelled internal standard was included, containing an equal mix of all samples in the experiment. Following gel running and scanning, image data was analyzed using the DeCyder™ software package (GE Healthcare, Uppsala, Sweden). Normalization was performed on the basis of the internal standard, facilitating comparison between different gels. Protein spots exhibiting statistically significant changes in abundance across the 14 gels in the experiment were identified using a student's t-test with applied false discovery rate (FDR) and a p-value threshold of p<0.05. Performing this analysis on light-adapted versus dark-adapted preparations resulted in the identification of 31 protein spots exhibiting statistically significant changes in abundance (figure 2). The gels were CBB stained, matching protein spots were excised and 30 spots were successfully identified by MALDI-TOF MS. Of the identified spots, 19 represented known lumen proteins while the remainder were stroma proteins or proteins of unknown localisation. 26 protein spots showed significantly increased levels and five spots significantly decreased levels in the light-adapted plants. The proteins showing changed abundance are presented in table 3 (lumen proteins) and table 4 (non-lumen proteins and unidentified protein spots). Four of the up-regulated proteins are known to be components of or related to PSII: PsbP1 (At1g06680), which is essential for the regulation and stabilization of PSII [19], showed a fold change of 1.43–1.48. PsbQ2 (At4g05180), one of two isoforms of PsbQ, which is required for PSII assembly/stability in low light conditions [20], displayed an increased abundance of 1.78–1.88 fold. The major form of plastocyanin (At1g20340) which transfers electrons from cytochrome-*f* to photosystem I (PSI), was up-regulated 1.46–1.54 fold. Finally HCF136 (At5g23120), a chaperone-like assembly factor, which displays a PSII-less phenotype in T-DNA knock out mutants [21], was up-regulated by a factor 1.53–1.57. Besides these four proteins, the TL26 PsbP-like protein (At3g55330, also referred to as PPL1) which is required for the efficient repair of photodamaged PSII [4] and eight additional lumen proteins of unknown or predicted function were identified as being up-regulated in the light-adapted preparation with fold changes ranging from 1.25–1.63. Two stromal proteins, the two isoforms of leaf FNR (ferredoxin-NADP^+^-oxidoreductase), AtLFNR1 (At5g66190) and AtLFNR2 (At1g20020), were up-regulated 1.51 and 1.47–1.52 fold respectively. Leaf FNR plays a well defined role in linear electron transport in chloroplasts, with the two isoforms forming homo- or heterodimers in complex with ferredoxin [22]. Finally two proteins of unknown sub-cellular localisation and function, At5g27390 and At5g42765, showing an up-regulation of 1.09 and 1.59 fold respectively, were identified. These two proteins both exhibit putative bipartite transit peptides and a twin arginine motif, indicating that they may be transported into the thylakoid lumen via the TAT-pathway. A recent mass spectrometry based study, identifying the probable N-terminal peptide, supports this hypothesis in the case of At5g42765 [23]. The At5g27390 protein is predicted to contain a PsbP-like domain, characteristic of members of the PsbP-like protein family known to reside in the thylakoid lumen [2]. On the other hand, both subunits A and B of stromal glyceraldehyd 3-phosphate dehydrogenase, which catalyse the reaction D-glycerate 1,3 bisphospate to glyceraldehyd 3-phosphate, using NADP as an electron carrier, are down regulated, as is carbonic anhydrase and sedoheptulose-bisphosphatase. An increase in levels of Bovine Serum Albumin (BSA), a contaminant from the preparation medium, was also observed in 8-h light-adapted samples. Upon light exposure the thylakoid membranes become stressed and it is probable that a slightly higher degree of thylakoid leakage takes place, leading to the observed abundance change. For this reason only lumen proteins displaying an increased abundance should be considered in this type of experiment, which in this study all are.

**Table 2:** DIGE experimental design for 16 h dark-adapted vs. 8 h light-adapted thylakoid lumen preparations.

Gel nr.	Cy 2	Cy 3	Cy 5
1	pooled standard	16 h dark-adapted A	8 h light-adapted A
2	pooled standard	8 h light-adapted B	16 h dark-adapted B
3	pooled standard	16 h dark-adapted C	8 h light-adapted C
4	pooled standard	8 h light-adapted D	16 h dark-adapted D
5	pooled standard	8 h light-adapted A	16 h dark-adapted A
6	pooled standard	16 h dark-adapted B	8 h light-adapted B
7	pooled standard	8 h light-adapted C	16 h dark-adapted C
8	pooled standard	16 h dark-adapted D	8 h light-adapted D
9	pooled standard	8 h light-adapted A, with NaBr	16 h dark-adapted A, with NaBr
10	pooled standard	16 h dark-adapted B, with NaBr	8 h light-adapted B, with NaBr
11	pooled standard	8 h light-adapted C, with NaBr	16 h dark-adapted C, with NaBr
12	pooled standard	16 h dark-adapted D, with NaBr	8 h light-adapted D, with NaBr
13	pooled standard	16 h dark-adapted A, with NaBr	8 h light-adapted A, with NaBr
14	pooled standard	8 h light-adapted B, with NaBr	16 h dark-adapted B, with NaBr
15*	pooled standard	16 h dark-adapted C, with NaBr	8 h light-adapted C, with NaBr
16*	pooled standard	8 h light-adapted D, with NaBr	16 h dark-adapted D, with NaBr

* Gels were not included in the DeCyder analysis due to scanning problems.

**Table 3:** Lumenal protein spots displaying significant changes in spot intensity. Thylakoid lumen and suggested thylakoid lumen proteins identified by MALDI-TOF MS exhibiting statistically significant changed relative protein levels in light-adapted as compared to dark-adapted Arabidopsis plants, determined by a t-test with applied FDR and a significance threshold of p<0.05. Positive average ratios represent a higher relative protein level in light-adapted plants.

Spot No.	Protein description	Gene locus (TIGR)	Average Ratio	T-test with FDR	Mascot score	Mass of precursor	Matched peptides	Sequence coverage
3	HCF136	At5g23120	1.57	0.043	132	44076	12	32%
4	HCF136	At5g23120	1.53	0.031	177	44076	17	45%
12	PsbP1	At1g06680	1.46	0.031	75	28078	8	45%
13	PsbP1	At1g06680	1.58	0.031	77	28078	9	39%
14	PsbP1	At1g06680	1.43	0.043	90	28249	8	39%
15	35.8 kDa PsbP-like	At5g11450	1.46	0.047	112	33344	11	38%
17	17.5 kDa PPIase	At2g43560	1.58	0.031	74	23549	6	21%
18	17.9 kDa unknown lumen protein	At4g24930	1.63	0.031	78	24680	7	28%
19	15.9 kDa PsbP-like	At1g76450	1.59	0.032	82	27473	7	38%
20	Unknown lumen protein	At5g42765	1.59	0.031	64	25083	6	26%
22	TL17 pentapeptide protein	At5g53490	1.48	0.040	114	25628	11	48%
23	TL16 unknown lumen protein	At4g02530	1.38	0.031	74	23648	7	31%
24	15.0 kDa unknown lumen protein	At5g52970	1.48	0.031	83	24553	7	19%
25	PLAT, Plastocyanin major	At1g20340	1.46	0.043	77*	10445	4	54%
26	TL15 pentapeptide protein	At2g44920.2	1.25	0.049	131	23764	11	55%
27	TL26 PsbP-like	At3g55330	1.56	0.031	137	25607	12	46%
28	Proposed lumen protein	At5g27390	1.09	0.031	102	26751	8	47%
29	PsbQ2	At4g05180	1.88	0.043	123	24628	11	49%
30	PsbQ2	At4g05180	1.78	0.043	136	24628	11	46%
31	PsbQ2	At4g05180	1.79	0.043	111	24628	9	46%

* Identified using an in-house modified Arabidopsis TAIR database.

**Table 4:** Non-lumenal protein spots displaying significant changes in spot intensity. Non-lumenal and unidentified protein spots displaying differential relative protein levels. Positive average ratios represent a higher relative protein level in light-adapted plants while negative ratios denote a lower relative level.

Spot No.	Protein description	Gene locus (TIGR)	Average Ratio	T-test with FDR	Mascot score	Mass of precursor	Matched peptides	Sequence coverage
1	Unidentified	-	1.97	0.031	-	-	-	-
2	BSA	gi|30794280	1.79	0.043	83	69278	13	25%
5	GAPDH subunit B	At1g42970	−1.35	0.047	151	47630	20	33%
6	Sedoheptulose-bisphosphatase	At3g55800	−1.32	0.046	86	42815	15	28%
7	Sedoheptulose-bisphosphatase	At3g55800	−1.32	0.047	138	42787	21	40%
8	GAPDH subunit A	At3g26650	−1.41	0.031	165	42463	16	36%
9	LEAF FNR 1	At5g66190	1.51	0.047	171	40301	21	54%
10	LEAF FNR 2	At1g20020	1.47	0.046	85	41142	15	26%
11	LEAF FNR 2	At1g20020	1.52	0.042	161	41142	23	63%
16	Carbonic anhydrase	At3g01500	−1.26	0.046	130	28166	17	50%
21	Unidentified**	-	1.54	0.032				

** According to location on gel identified as Plastocyanin major (At1g20340) [40].

### Co-expression analysis of lumen protein genes

As previously stated analysis of public microarray data using the Arabidopsis eFP Browser (bar.utoronto.ca, Short Day series, [17], [18]) revealed that mRNA levels for the majority of known lumen proteins were elevated in response to light according to a diurnal pattern. The experimental data, using the DIGE technique, indeed found thirteen lumen proteins, ∼35% of the lumen proteome experimentally identified by [2], as being up-regulated in light-adapted as compared to dark-adapted Arabidopsis plants. Although this gives an indication of common transcriptional control for the lumen proteome, regulated by light or circadian rhythm, it was interesting to investigate if the lumen protein genes were in fact more generally co-expressed, both across developmental stages and tissue types as well as by different stimuli. To study this, co-expression analysis using the Arabidopsis Co-expression Tool (http://www.arabidopsis.leeds.ac.uk/act/, experiments 2_1-2_50, [24]) was performed on array probes corresponding to not only the genes coding for lumen proteins identified by the proteomics analysis but also on probes for all other previously experimentally identified lumen protein genes according to [2].

**Figure 3:**
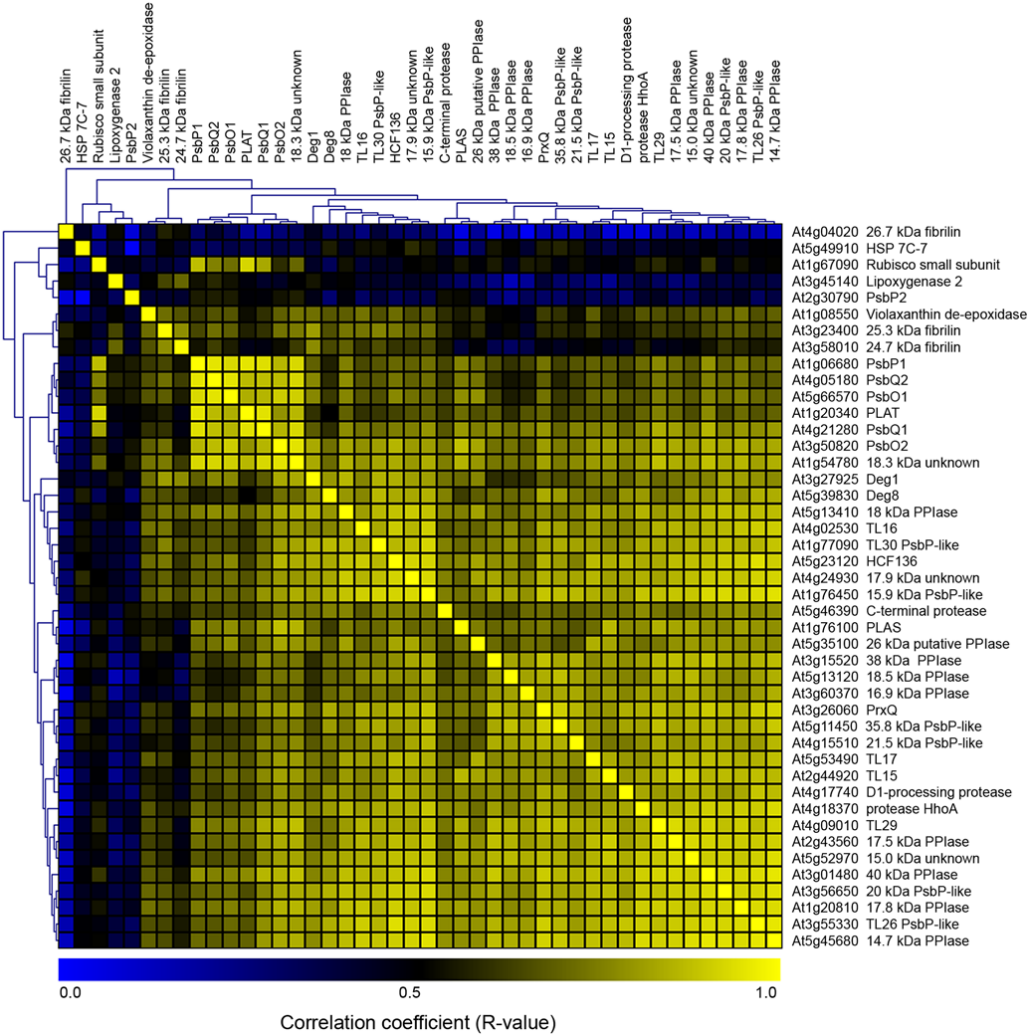
Hierarchical clustering of co-expression coefficients for thylakoid lumen protein genes and typical stroma contaminant genes. Co-expression correlation coefficients (r-values) were extracted from the Arabidopsis Co-expression database and subjected to hierarchical clustering. A high level of co-expression is indicated by yellow color while blue color represents very low co-expression. While stroma protein genes display low co-expression to lumen protein genes, lumen protein genes exhibit very high levels of co-expression among each other.

When using almost any given lumen protein gene as a query gene for the multi-experiment co-expression database tool, the resulting list of co-expressed genes contained many other genes coding for lumen proteins with high correlation coefficients (data not shown). To study the extent of this co-expression among the lumen protein genes in detail, co-expression correlation coefficients between all lumen protein genes were extracted from the database and subjected to unsupervised hierarchical clustering and visualised as a heat map (figure 3). Three genes encoding typical stromal contaminants; HSP 7C-7, Lipoxygenase 2 and the small subunit of RUBISCO were included in the analysis, as well as three genes encoding fibrilins previously identified in lumen preparations but where the exact localisation remains unconfirmed [2]. High co-expression correlation coefficients (r-values), typically in the range of 0.80 to 0.95, were observed between the majority of lumen protein genes. Exceptions were the genes encoding violaxanthin de-epoxidase and PsbP2, which displayed low co-expression to other lumen protein genes. This could be an effect of either distinct differences in function or of low transcript abundance. A prominent cluster was identified, consisting of genes encoding the extrinsic PSII proteins together with genes encoding the major form of plastocyanin (PLAT) and an 18.3 kDa lumen protein recently implicated in the regulation of the PSII repair cycle [5]. These seven genes showed very high correlation coefficients (r-values around 0.90 and above) among each other but lower towards other lumen protein genes. Among the remaining genes several interesting clusters could be observed. The genes encoding the serine proteases Deg1 and Deg8 clustered together, while the gene encoding Deg5 (HhoA) clustered alone. This is interesting because Deg5 and Deg8 are believed to form hexameric complexes in the lumen [25]. On the other hand Deg1 and Deg8 are proteolytically active towards photodamaged D1-protein *in vitro*, which Deg5 appears not to be [25], [26]. Furthermore two genes, encoding the pentapeptide proteins TL15 and TL17, clustered together. Although the function of these proteins remains unknown, three out of four of the protein family members in Arabidopsis are known or predicted to be located in the thylakoid lumen [27]. Finally a cluster of genes encoding immunophilin proteins (38 kDa PPIase, 18.5 kDa PPIase and 16.9 kDa PPIase) was observed. While the 16.9 kDa PPIase has been implicated in the accumulation of PSII supercomplexes [28], the precise function of the other lumenal immunophilins remains unknown. The genes encoding stromal contaminants exhibited low (r<0.50) correlation coefficients to the genes coding for lumen proteins, although the gene encoding the small subunit of RUBISCO shares a certain amount of co-expression with genes coding for the oxygen evolving complex (OEC) proteins of PSII and the major form of plastocyanin. The genes for the light-induced lumen proteins identified by the DIGE experiments were spread among the different co-expression clusters, displaying, with the exception of those encoding PsbP1, PsbQ1 and major plastocyanin, high correlation coefficients to other lumen protein genes.

It is evident from this analysis that the majority of lumen protein genes have very similar expression profiles across the microarray experiments included in the Arabidopsis Co-expression Tool database (http://www.arabidopsis.leeds.ac.uk/act/, experiments 2_1-2_50, [24]), indicating that their regulation at the transcriptional level is very similar, irrespective of the environmental condition, developmental stage or tissue type. This in turn suggests common function or participation in common biological pathways for these gene products. Based on this observation it seemed reasonable to assume that novel lumen related proteins could be identified by co-expression analysis. Co-expression correlation coefficients between the known lumen protein genes and all 22000 genes represented on the Affymetrix ATH1 arrays were extracted from the Arabidopsis Co-expression Tool and combined to a single dataset. Genes co-expressing specifically with the prominent cluster identified in figure 3 (containing genes encoding the lumenal extrinsic PSII components, the major form of plastocyanin and the 18.3 kDa lumen protein) were identified by calculating their mean correlation coefficient against the seven genes in the cluster. As expected the list of genes displaying the highest mean r-values to the cluster is dominated largely by genes encoding other components of PSII as well as genes encoding components of PSI (supplemental data, table S1). The remaining lumen protein genes, which showed a lower degree of co-expression with the genes in the above mentioned cluster but a high degree of co-expression among themselves (genes to the right of the gene encoding the 18.3 kDa protein with the exception of the gene coding minor plastocyanin (PLAS) in figure 3), were used in the same way to identify novel lumen related proteins. Genes exhibiting mean r-values higher than 0.8 were selected in order to obtain a subset of 176 genes with high co-expression to the majority of lumen protein genes (the top 60 genes are shown in supplemental data, table S2). The subset is to a large extent comprised of genes of unknown or putative function although there is an interesting representation of genes encoding ribosomal proteins and redox-signalling proteins, including several members of the thioredoxin family. Strengthening the hypothesis that these gene products may be related to the thylakoid lumen, TargetP predicts 89% of the gene products as being chloroplast targeted. Also 87% are reported in the Plant Proteome Database (http://ppdb.tc.cornell.edu/, [29]) as having a plastid localisation. One of the unknown proteins identified in the proteomic part of this study, encoded by the gene At5g42765, is among the highly co-expressed genes, with a mean co-expression correlation coefficient of 0.85, suggesting further that it is a true lumen protein as reported in another study [23]. Also an immunophilin (At3g10060) and Psb27 (At1g03600), experimentally shown to be lumen proteins [3], have high correlation coefficients of 0.83–0.85.

In summary the results from this study show that a major portion of the lumen proteome exhibits increased protein expression in light-adapted as opposed to dark-adapted Arabidopsis plants. Proteins displaying light-induced expression included the PsbP1 and PsbQ2 extrinsic components of PSII, the major form of plastocyanin and several other lumen proteins of various or unknown function. Furthermore co-expression analysis indicates that the lumen proteome encoding genes are transcriptionally regulated in a common manner, not only as a response to light, but uniformly across stress conditions, developmental stages and tissue types.

## Discussion

Plants sense light of different types by a set of specific photoreceptors distinct from the pigments of photosynthesis. The perceived light induces signalling cascades, resulting in short-term responses as well as long-term acclimation. Besides the direct perception of light by photoreceptors, plants also sense light via plastid redox-sensing mechanisms and possess an internal clock system regulating gene transcription, the circadian clock, which runs on a period of 24 hours. Rather than being separate processes, light signalling pathways and circadian control are interconnected pathways, with photoreceptors being involved in timing of the clock, while the clock regulates expression of photoreceptor genes (for review see [30]). It has been predicted that more than 6% of Arabidopsis genes are under circadian control and that as many as 30–50% of genes expressed in rosette leaves undergo some form of diurnal expression changes [8], [9]. One prominent group of genes displaying circadian control identified by Harmer et al. [8] encodes 22 components of photosynthesis, including *LHCAs*, *LHCBs* as well as PSI and PSII reaction centre genes. All these genes exhibit co-regulation, with mRNA levels peaking around midday. In a second study [9] it was shown that ∼23% of photosynthesis genes undergo diurnal expression changes of an amplitude higher than 1.75-fold. Light-regulation of nuclear photosynthesis genes is well known [31], but in depth it is a complex interplay of photoreceptor-, circadian-, redox- and sugar-mediated signalling pathways.

Considering the recent increase in our knowledge regarding the involvement of several thylakoid lumen proteins in the regulation of photosynthesis [4], [5], [19], [20], [28], [32], it was interesting to study how and to which extent these proteins are differentially expressed during the day and night. A study of publicly available microarray data indicated that the majority of genes coding for lumen proteins seemed to undergo diurnal expression changes, with expression peaking during the light period (Arabidopsis eFP browser, bar.utoronto.ca, Short Day series, [17], [18]). To study the extent of this regulation in more detail at the protein level, we compared protein expression between the thylakoid lumen proteomes of 8 h light-adapted and 16 h dark-adapted Arabidopsis plants using the DIGE technique. We identified thirteen lumen proteins undergoing significant abundance changes, showing an approximate increase of up to 1.9-fold after the 8 h light treatment. Especially noteworthy is that three of these proteins, PsbP1, PsbQ2 and major plastocyanin, are among the most highly abundant lumen proteins [2].

The presence of the extrinsic PSII subunits PsbP1 and PsbQ2 among the up-regulated lumenal proteins is consistent with the previous observation that the chloroplast lumen contains a soluble pool of unbound extrinsic PSII proteins that may serve as a ready source of material for the assembly of new OECs, being directly available after D1-turnover and PSII re-assembly [33]. Consistent with our observed up-regulation of the PsbP1 and PsbQ2 proteins this would be especially important during the light period, when the rate of D1-turnover increases with light intensity [34]. Intriguingly though, we could not discern any change in levels of PsbO protein. Possibly an increased protein expression was masked by a simultaneous increase of PSIIs to which PsbO became bound. The major form of plastocyanin, another critical lumenal component of the photosynthetic electron transport chain, which functions in electron transport from cytochrome-b_6_ *f* to PSI, also displayed significant up-regulation of protein levels in the light. The plastocyanin promoter has been extensively studied previously and it is well known that gene expression is induced by light, via an interplay of photoreceptor- and redox-signalling [35], [36]. Together with these well studied components of photosynthesis, HCF136, a protein required for PSII assembly and/or stability, was up-regulated in light-adapted plants [21], as was the TL26 PsbP-like protein, required for efficient repair of photodamaged PSII [4]. Two other PsbP-like proteins of unknown function were also identified, but although they contain a similar domain as the TL26 PsbP-like protein, they may be involved in distinctly different processes, as demonstrated recently in the study by Ishihara and co-workers [4]. Of the other up-regulated lumen proteins very little is known regarding their function and possible relation to photosynthesis. Further characterization of these proteins in detail is an exciting area of future research, possibly leading to the identification of novel factors involved in the regulation of photosynthesis. Although it is probable that most of the observed changes in protein abundance are due to changes in protein expression it cannot be ruled out that some changes may also be partly caused by increased/decreased translocation across the thylakoid membrane and changes in the strength of interactions between proteins and the thylakoid membrane.

The proteomic analysis showed that a large portion of the lumen proteome was under some form of diurnal regulation, hinting at a certain level of co-expression. But because as much as 30–50% of expressed genes in Arabidopsis rosette leaves are expected to undergo diurnal expression changes [9], the high level of co-expression between lumen protein genes, as observed by co-expression analysis, cannot be attributed to light/dark-cycle dependent expression alone. While the proteomic analysis only studied light/dark induced changes of protein levels, co-expression analysis in contrast considers transcriptional expression in very many different experimental conditions. Performing this type of analysis showed that transcription of the prevalent lumen protein genes is similarly regulated across different environmental conditions, tissue types and developmental stages. Clustering of co-expression data revealed that although almost all lumen protein genes share a high level of co-expression, certain sub-groups could be identified. Most prominently, a cluster containing genes encoding the OEC proteins, major plastocyanin and the 18.3 kDa lumen protein was observed. The inclusion of the 18.3 kDa lumen protein gene in this tight cluster is consistent with and strengthens the suggestion that the protein is intimately involved in regulation of PSII [5]. Clustering of the genes encoding two pentapeptide proteins and of a group of immunophilins, suggests that these related proteins may also share common function in the lumen. Expanding the co-expression analysis to include all other genes in the genome showed that the OEC encoding gene cluster co-expressed with genes for other PSII and PSI components. This proved the robustness of the analysis approach, hence suggesting that it also could be used to identify novel genes co-regulated with the other lumen protein genes. A well known problem of transcriptomic analysis though, is genes expressed at low levels, which probably is the case for so far unidentified lumen protein genes. A detailed analysis of common regulatory elements in the promoters of the genes for the lumen proteins is necessary to further understand the mechanisms controlling the observed uniform regulation.

Taken together the present study shows that ∼35% of the prevalent lumen proteome displays increased abundance after exposure to 8 hours of light. This includes both proteins involved directly in photosynthesis as well as lumen proteins of unknown function. Furthermore we conclude from co-expression analysis of known lumen protein genes that transcriptional regulation of these genes occurs uniformly, a prospect which facilitated the identification of a variety of chloroplast protein encoding genes sharing high co-expression with the lumen protein genes. Further studies regarding the origin and consequences of the observed increases in protein levels, together with detailed characterization of lumen proteins of still unknown function are now necessary in order to further understand the biological role that the light-induced abundance changes play.

## Materials and Methods

### Plant material

*Arabidopsis thaliana* ecotype Columbia were grown on soil for 7 weeks with a dark/light cycle of 16/8 hours using a light intensity of 120–150 μmol photons m^−2^ s^−1^. For the fast lumen preparation leaves were harvested before the light was turned on (16 h dark-adapted plants) and before the light was turned off at the end of the 8 h light period (8 h light-adapted plants).

### Thylakoid lumen preparation

After harvesting the leaves were kept in ice cold water in either light or darkness for 20 minutes before the preparations were started. All preparation was performed at 4°C and dark-adapted leaves were harvested and prepared under green light. Arabidopsis chloroplasts were prepared from 60 g leaves, divided in portions of 10 g which were blended in 200 mL of homogenising buffer (20 mM Tricine-NaOH (pH 8.4), 300 mM sorbitol, 10 mM EDTA, 10 mM KCl, 0.25% (w/v) bovine serum albumin (BSA), 90 mM sodium ascorbate, and 5 mM cysteine) five times for 1 sec. using a Heidolph DIAX 900 homogeniser. The preparation continues as described in [13] with changes for *Arabidopsis thaliana* thylakoid lumen preparation according to [2]. Thylakoid membranes were washed twice with 10 mM sodium pyrophosphate (pH 7.8), twice with 300 mM sucrose in 2 mM Tricine (pH 7.8) and twice using fragmentation buffer (pH 7.8). Between washes thylakoids were resuspended and homogenised in a glass homogeniser and centrifuged for 5 min at 7500×g. All according to [13] with the exception that 1 mM EDTA was added to the last fragmentation buffer before Yeda press fragmentation of the thylakoids. Thylakoid membrane fragments were ultra-centrifuged at 200 000×g for 60 min. Supernatants (lumen fraction) were moved to new tubes followed by an additional centrifugation step.

In the fast thylakoid lumen preparation, the chloroplast preparation was performed as above. The washing steps of the thylakoid membranes with sodium pyrophosphate, sucrose and one of the wash steps with fragmentation buffer were excluded before Yeda press fragmentation. The first ultra-centrifugation was performed for only 10 min at 200 000×g and after discarding the thylakoid membrane fragment a new ultra-centrifugation for 1 hour was performed. Four fast thylakoid lumen preparations of day- and four preparations of night-acclimated plants were made from plants in the same development stage. Fast lumen preparation with the addition of 5 mM NaBr in the fragmentation buffer was also used to extract more of the proteins loosely bound to the thylakoid membrane. However, this modification did not have any significant effect on the composition of the lumenal fractions obtained.

### Concentration of protein samples and protein assay

The lumen fraction was concentrated using Microsep 3K centrifugal devices (PALL Life Ccience) and the buffer was diluted with double distilled water approximately to half the strength in order to decrease the salt concentration prior to iso-electric focusing (IEF). Protein quantification was carried out according to the method of Bradford [37] using bovine serum albumin (fraction V) as a standard. The method was adjusted to a smaller amount for use with ELISA plate wells; 5 μL of standard BSA solution or sample and 195 μL of room temperature Bradford solution were measured at 595 nm. The chlorophyll content was determined according to [38].

### Difference gel electrophoresis (DIGE)

Lumen samples were precipitated with 4 volumes of ice cold acetone overnight at −20°C and the precipitated lumen proteins were collected by centrifuged at 12 000×g for 15 minutes at 4°C. The supernatant was removed and the pellet air dried for 5 minutes. The lumen proteins were solubilised and labelled according to the GE Healthcare manual for DIGE (GE Healthcare, Uppsala, Sweden), with the exception that 400 pmol CyDye colour to 100 μg of protein was used. Samples were labelled with Cy3 and Cy5 while an internal standard containing a mix of equal amounts of the two samples was labelled with Cy2. Tables 1 and 2 show the experimental designs used. The final concentration of solubilisation buffer was; 7.4 M urea, 1.1 M thiourea, 1.3 mM tris, 4% CHAPS, 76 mM DTT and 0.8 % IPGbuffer (pH 3–11 NL) (GE Healthcare, Uppsala, Sweden). 63 μg of protein (21 μg of each CyDye labelled sample and 21 μg of Cy2 labelled internal standard) in 450 μL of the solubilised and labelled thylakoid lumen sample was applied to 24 cm immobiline strips, pH 3–11 NL (GE Healthcare, Uppsala, Sweden), passive rehydrated for two hours and active rehydrated at 60 V for 10 hours. After rehydration the strips were moved to a 12-manifold and iso-electric focusing was performed on an IPGphor II (GE Healthcare, Uppsala, Sweden). Strip equilibration and second dimension electrophoresis were performed according to the manufacturer's instructions (2D- Electrophoresis - Principles and Methods. GE Healthcare, Uppsala, Sweden) as follows: Equilibration was performed in 20 mL standard equilibration solution for 15 minutes with 2% DTT and 15 minutes with 4.5% iodoacetamide. All strips were washed with 2×electrophoresis buffer prior to loading on the second dimension gel and sealed with 2×electrophoresis buffer containing 0.5% low melting agarose. The second dimension was run on 12–20% SDS-polyacrylamide gradient gels using an Ettan Dalt*six* electrophoresis unit. Prior to gel casting the glass plates were coated with bind-silane solution in order to keep the gels fixed to one glass plate during post staining and automatic spot picking (2D- Electrophoresis - Principles and Methods, GE Healthcare, Uppsala, Sweden).

### Gel imaging and analysis

Labelled proteins were visualised using a Typhoon™ 9400, Variable Mode Imager, (GE Healthcare), scanned in-between low fluorescence glass-plates (GE Healthcare). The three Cy colours were scanned with recommended filters according to the DeCyder manual as follows; Cy2 images were scanned using a 488 nm laser and a 520 nm band pass (BP) 40 emission filter (EF), Cy3 images were scanned using a 532 nm laser and a 580 nm BP 30 EF and Cy5 images were scanned using 633 nm laser and a 670 nm BP 30 EF. All gel images were scanned at 100 μm resolution and the photo-multiplier tube was set to ensure maximum pixel intensity without saturated spots. Prior to gel analysis the image extraneous areas were removed using ImageQuant™ V5.2 (GE Healthcare). Gel analysis was performed using DeCyder™ V 6.5 (GE Healthcare), designed specifically to be used for DIGE. The estimated number of spots used in the batch processor was set to 2500, as recommended, and no exclusion filter was applied before processing. The DeCyder™ statistical tool was used to calculate average ratios by a t-test with applied false discovery rate (FDR) and a significance threshold of p<0.05. Prior to spot picking the gels were hot Coomassie stained according to the method of Reiner Westermeier and Tom Naven [39]. Spots were picked using an Ettan Spotpicker™ with a 1.4 mm picker head (GE Healthcare, Uppsala, Sweden).

### In-gel digestion and MALDI-TOF MS

Gel pieces were dehydrated and de-stained with 35% acetonitrile in 20 mM ammonium hydrogen carbonate, three times 30 minutes. Complete drying of the gel pieces was performed by addition of 100% acetonitrile for 5 minutes two times. The dry gel pieces were digested with 3–5 ng/μl trypsin (Promega) in 20 mM ammonium hydrogen carbonate and 10% acetonitrile over night. In-gel digested proteins were analysed with a MALDI-TOF Voyager-DE™ STR Bio Spectrometry™ Workstation from Applied Biosystems. Database searches were performed on an in-house Mascot server licensed to Umeå University by Matrix Science (http://www.matrixscience.com) using the NCBInr, Arabidopsis TAIR7 databases and an in-house modified Arabidopsis TAIR7 database where experimentally determined mature lumen protein sequences are included. The search parameters were set to allow an error for peptide masses of 50 ppm and one missed cleavage site. Oxidation of methionine and carbamidomethylation of cysteine were set as parameters for variable modification.

### Co-expression analysis

Co-expression correlation coefficients (r-values) between Affymetrix probes corresponding to genes encoding all experimentally identified thylakoid lumen proteins described by [2] as well as three selected typical stromal contaminants were extracted from the Arabidopsis Coexpression Data Mining Tools (www.arabidopsis.leeds.ac.uk/act/, experiments 2_1-2_50, [24]). The resulting data matrix, containing all permutations of co-expression coefficients for the 44 genes, was clustered by unsupervised hierarchical clustering using the MultiExperiment Viewer software v.4.2 (MeV) from The Institute for Genomic Research (J. Craig Venter institute), (www.tm4.org/mev.html). The clustered data was visualised as a heat map with the r-value colour scale set from 0 (no correlation) to 1 (perfect correlation). Co-expression coefficients between a lumen protein gene probe and probes representing all genes on the Affymetrix ATH1 array were extracted using the multi-experiment co-expression tool of the Arabidopsis Coexpression Data Mining Tools. This process was repeated for all the lumen protein genes described above. In the resulting data matrix, each probe on the Affymetrix ATH1 array is thereby represented by 38 co-expression correlation coefficients corresponding to the probes co-expression with all lumen protein gene probes. In order to identify genes showing high co-expression with selected lumen protein genes the mean correlation co-efficient for the selected lumen protein genes and any given probe was calculated. Ranking of the probes according to the mean correlation co-efficient was then used to determine which genes were specifically co-expressed with the identified PSII-related cluster and to the remaining lumen protein genes. The experiments in the database in total represented 422 arrays and details regarding each microarray experiment can be easily obtained at www.arabidopsis.leeds.ac.uk/act/expinfo.php.

## Supporting Information

Table S1 Genes co-expressed with lumenal PSII genes. 30 genes sharing the highest co-expression with a cluster of lumenal PSII genes identified in figure 3. Mean correlation co-efficients (r-values) were calculated from the r-values between a gene and each gene in the lumenal PSII cluster.
(0.03 MB XLS)

Table S2 Genes co-expressed with 29 selected lumen protein genes. 60 genes sharing the highest co-expression with 29 selected lumen protein genes identified as exhibiting a high level of co-expression. Mean correlation co-efficients (r-values) were calculated from the r-values between a gene and each of the 29 selected lumen protein genes.
(0.04 MB XLS)
